# Total Hip Arthroplasty for Osteonecrosis of the Femoral Head: A Mid-term Follow-Up in Patients From Northern India

**DOI:** 10.7759/cureus.70360

**Published:** 2024-09-27

**Authors:** Naman Parakh, Amit Saraf, Sandeep Bishnoi, Santosh Kumar Singh, Divyam Jindal, Shivam Madan

**Affiliations:** 1 Orthopaedics, Teerthanker Mahaveer Medical College & Research Centre, Moradabad, IND; 2 Orthopaedics and Traumatology, Maa Vindhyawasini Autonomous State Medical College, Mirzapur, IND; 3 Orthopaedics, Dayanand Medical College and Hospital, Ludhiana, IND

**Keywords:** complication, functional and radiological outcome, haris-hip score, osteonecrosis of femoral head, total hip athroplasty

## Abstract

Introduction: Osteonecrosis of femoral head (ONFH) is a disabling condition of the hip joint with multifactorial etiology and is associated with genetic predilection and exposure to certain risk factors most commonly being chronic alcohol or steroid intake. Total hip arthroplasty (THA) remains the gold standard for end-stage femoral head osteonecrosis. The outcome after the surgery is mainly affected by age, activity levels and other factors that affect the development of ONFH. Through this study we aimed to evaluate the clinical and radiological outcomes in the patients who have undergone uncemented THA for ONFH.

Materials and methods: We included 111 uncemented THA on 84 patients with ONFH done by a single surgeon in our institution. The patients were followed up postoperatively and were evaluated based on their functional outcome using Harris hip scores (HHS) and the radiological outcome by serial radiographs on every follow-up.

Results: The mean preoperative HHS was 49.30 which showed significant improvement in the final follow-up to 96.17. The mean acetabular inclination and anteversion at final follow-up radiographs were 47.70 and 18.67 degrees respectively. Fifteen patients had complications which included post-operative surgical site infection (three), hip pain (six) and thigh pain (six). Complications like osteolysis, poly wear and femoral subsidence were recorded in a total of six patients at the final follow-up. Three patients underwent revision THA due to increased femoral subsidence, accelerated poly wear, and aseptic loosening due to osteolysis.

Conclusion: Our current observational study, which included 84 patients and 111 hips affected by osteonecrosis of the femoral head, examined the outcomes of total hip arthroplasty using metal on ultra-high molecular weight polyethylene liners. The results showed excellent clinical and radiological outcomes at a mean follow-up period of 4.8 years, particularly in younger patients with a mean age of 37.05 years.

## Introduction

Osteonecrosis of the femoral head (ONFH) is a devastating disease of the hip joint and becoming a huge burden of disease for the people of the Indian subcontinent in their young adulthood. It is a chronic, progressive, multi-factorial disease that ranges from its early stages of simple hip pain to advanced stages of complete hip joint destruction, which left them living crippled life. It may be caused due to exposure to certain risk factors which include chronic steroid and/or alcohol intake, smoking, along with various other chronic diseases [1]. A wide array of treatment options is available worldwide, from conservative ones in the form of various pharmacological agents to surgical options like core decompression, osteotomies and the most recent addition being the use of bone marrow mesenchymal stem cells and endothelial progenitor cells [2,3]. All these treatment modalities are focused on preserving the less damaged native hip joint. Despite immense hard work and efforts, no single treatment has proved to be promising in terms of a complete cure for the disease. Hence, the disease progresses to its end stages of joint destruction, leading to chronic disability.

Total hip replacement (THR) is the most used and reliable treatment option for reducing pain and restoring normal functional status in patients with advanced stages of ONFH. These patients are relatively younger, in their productive age group with high functional demands and need optimal restoration of their hip joint biomechanics closest to the native one [4]. Several techniques like preoperative templating and intraoperative use of anatomical landmarks ensure perfect component positioning for better functioning of the reconstructed joint [5].

The advent and availability of various government-funded health schemes has proven to be a boon to the population of lower socioeconomic backgrounds and has provided them the benefit and right to receive appropriate health care for a wide range of medical conditions.

The present study was conducted to assess: 1) potential aetiologies, 2) survivorship, 3) potential complications and 4) functional and radiological outcomes in the patients who underwent uncemented THR for ONFH.

## Materials and methods

The present study was a hospital-based, prospective, single-center, observational study conducted between January 2018 and December 2019 at a tertiary care center after clearance from the College Research Committee (CRC) and the Institutional Ethical Committee (IEC) of Teerthanker Mahaveer University, Moradabad, Uttar Pradesh, India (TMU/IEC/2024-25/004/11). A minimum of four-year follow-up was completed by January 2024. Eighty-four adult patients diagnosed with osteonecrosis of the femoral head (Grades 3 and 4) using the Ficat and Arlet staging system [6] who underwent uncemented total hip arthroplasty were included in the study. All included patients were operated under the government-funded health schemes. The conventional radiographs were used to screen, diagnose and stage the disease and Magnetic Resonance Imaging (MRI) of the involved hip/hips was performed to confirm the diagnosis and to exclude any other infective or inflammatory pathology. Written and informed consent were taken from all patients participating in the study.

Pre-operative protocol

All the patients included in the study were planned for uncemented THA and 100% magnified standard anteroposterior (AP) radiographs of the pelvis with a true lateral view of the affected hip were taken for each patient. Pre-operative Harris hip scores (HSS) were calculated as a reference for functional status [7]. Pre-operative templating was done on the radiographs for optimal sizing, positioning, and corrections to address the limb length discrepancies (LLD). Baseline and demographic characteristics of all patients were noted along with the identified etiology of ONFH.

Surgical procedure

The patients were operated on, under combined epidural/spinal anesthesia and a third-generation cephalosporin was administered intravenously as a standard protocol. All patients were operated on by a single surgeon using the posterior (Southern Moore) approach (mean incision length 12 cm) in a lateral position. Limb lengths and stability of the hip joint were checked manually. Metal-on-ultra high molecular weight polyethylene (UHMPE) Latitud implants (metal bearing surface with metaphyseal fitting) were used from the same manufacturer (Meril Life Sciences, Vapi, India). The metal-on-polyethylene (MoP) implant was chosen according to the allowances under the health care packages provided by the government-funded health schemes.

Post-operative protocol and follow-up

Immediate post-operative radiographs were done and all the parameters (acetabular component position like cup inclination and anteversion, femoral stem position, and LLD) were measured by two observers and the average was tabulated to avoid observer bias. First wound inspection and drain removal were done on the second post-op day. Patients were advised for full weight-bearing walk with the help of a walker from day two and kept on rehabilitation for ankle pumps, gait training, quadriceps and abductor strengthening exercises as tolerated. Suture removal was done at two weeks after surgery on an OPD basis depending upon the wound condition. Follow-up was done postoperatively at six weeks, six months, 12 months and yearly thereafter for clinical, functional and radiological evaluation.

Clinical and functional evaluation

This included a physical examination comprising the assessment of complications (early or late) if any- deep infection, anterior thigh pain, hip pain, any episode of instability or dislocation, LLD and gait abnormality. Functional assessment was performed by analyzing HHS [7], with the results being classified as excellent (90-100 points), good (80-89 points), fair (70-79 points), or poor (<70 points).

Radiological evaluation

**Figure 1:**
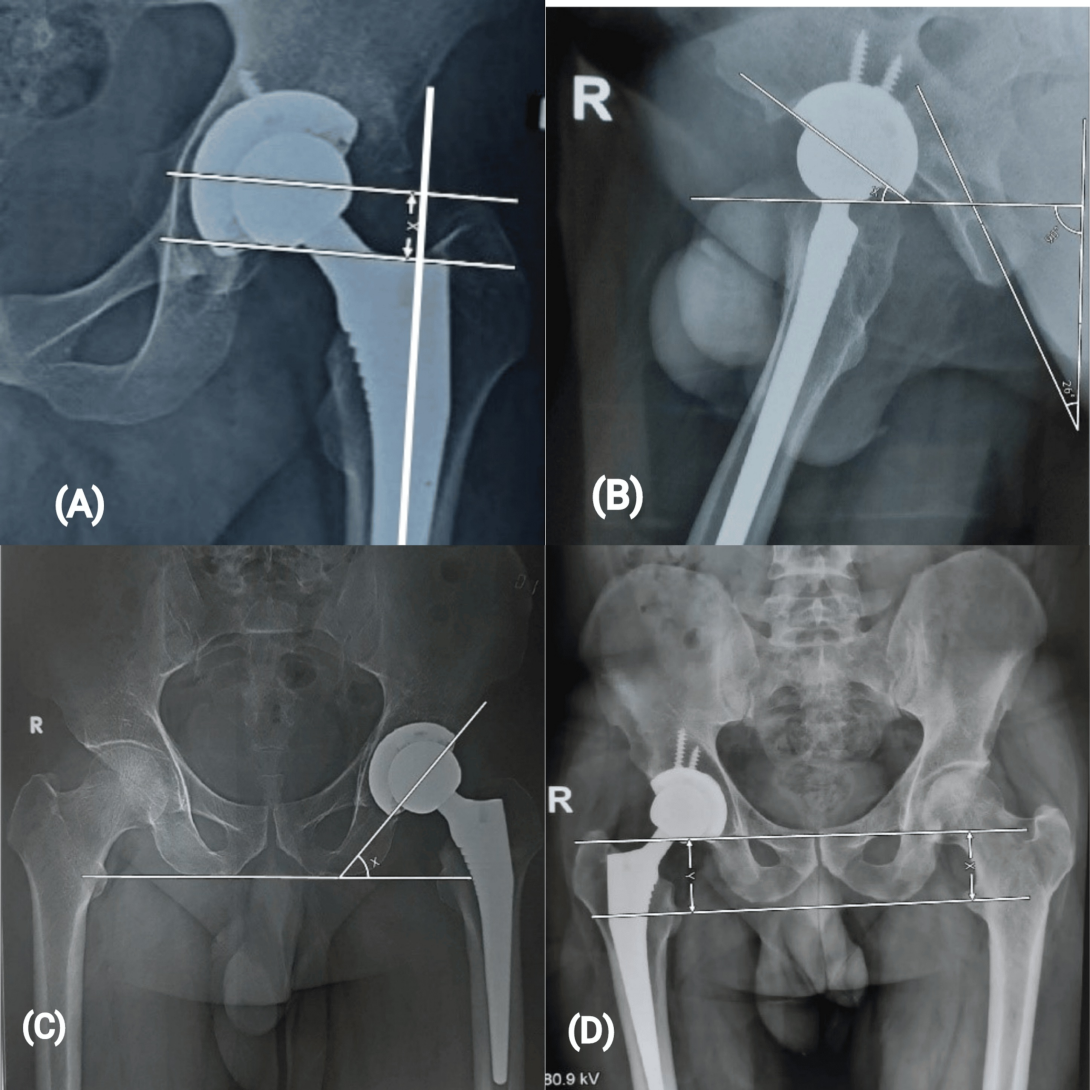
Measurement of (A) Femoral subsidence (x) on anteroposterior (AP) radiograph; (B) Acetabular anteversion (angle x) on cross table lateral radiograph; (C) Acetabular cup inclination (angle x) on AP radiograph; (D) Limb length discrepancy (LLD) (difference between lengths of x and y) on AP radiograph of pelvis

Standard radiographs included a true AP view of the pelvis and true lateral views of the hip and proximal part of the femur. Radiographs taken immediately postoperatively served as the baseline for all subsequent comparisons. The radiological evaluation of all parameters was done at each follow-up along with femoral stem subsidence along with evidence of polyethylene wear, osteolysis (septic/aseptic loosening) and implant migration. LLD, femoral subsidence and acetabular inclination were measured on a true AP radiograph of bilateral hips with pelvis. The acetabular anteversion was measured on a cross-table lateral view of the involved hip (Figure 1). Polyethylene wear, implant loosening (septic/aseptic) and evidence of cup migration were also evaluated.

Statistical analysis

The data so collected were tabulated in an Excel sheet (Microsoft, Redmond, WA, USA). The means and standard deviations of the measurements per group were used for statistical analysis (SPSS version 22.00 for Windows, IBM Corp., Armonk, NY, USA). For each assessment point, data were statistically analyzed using one-way ANOVA with Tukey's honestly significant difference (HSD) post hoc test. The difference between the two groups was determined using t-test as well chi-square test and the level of significance was set at p <0.05.

## Results

The study was carried out on 111 hips of 84 patients who underwent uncemented THR for ONFH for Grades 3 and 4 according to the Ficat and Arlet classification. The mean follow-up of the study was 4.8 years (range being four to six years). The gender distribution in the patients of ONFH in our study showed a male preponderance i.e., 71.43% of males (60 out of 84) and 28.57% of females. The age distribution showed most of the patients in the fifth decade (39 out of 84) of life with a mean age of 37.05 years and 73% of the patients were found to be of Grade 4 ONFH (81 out of 111). 32.14% of the patients had bilateral hip involvement (27 out of 84) whereas in unilateral cases, 33 patients (39.29%) had left hip involvement and 24 had right hip involvement (28.57%). The mean BMI in the study population was 21.48. The probable cause of ONFH in most patients was long-term steroid intake (57%) followed by idiopathic (29%) and chronic alcohol intake (14%). A total of 15 patients reported complications of hip pain (six), thigh pain (six) and surgical site infection (three) (Table 1).

**Table 1:** Characteristics of the study population AVN- Avascular Necrosis

SUBGROUP	NUMBER OF PATIENTS	%
GENDER		
Male	60	71.43
Female	24	28.57
AGE GROUP (IN YEARS)		
21-30	12	14.28
31-40	21	25
41-50	39	46.44
51-60	12	14.28
AVN GRADE		
3	30	27.03
4	81	72.97
SIDE		
Left	33	39.29
Right	24	28.57
Bilateral	27	32.14
AETIOLOGY		
Idiopathic	24	28.57
Steroid intake	48	57.14
Chronic alcohol intake	12	14.29
COMPLICATIONS		
Hip Pain	6	7.14
Surgical Site Infection	3	3.57
Thigh Pain	6	7.14

**Figure 2:**
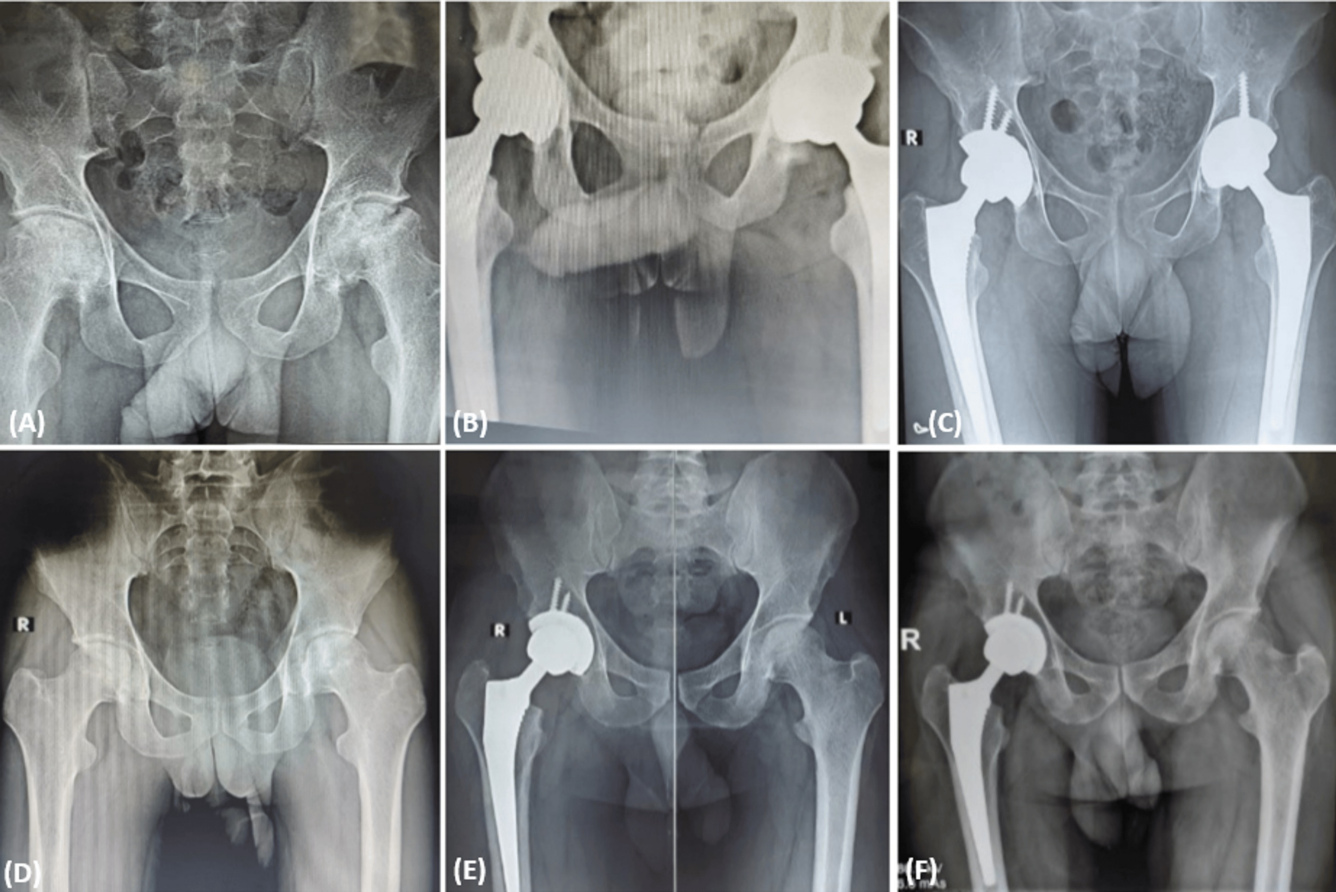
Patient 1- (A) Bilateral ONFH Stage Ⅳ left side and Stage Ⅲ right side; (B) Postoperative radiograph after bilateral uncemented THA; (C) Five-year follow-up showing well-fixed implants in bilateral hips with good functional outcome and no reported complication. Patient 2- (D) Bilateral ONFH Stage Ⅲ right side and Stage ⅡA left side; (E) Managed with uncemented THA for right side and core decompression for left side; (F) Four-year follow-up radiograph showing well-fixed components in the right hip with successful preservation of the native left hip joint with good functional outcome and no reported complication ONFH: Osteonecrosis of femoral head; THA: Total hip arthroplasty

The mean preoperative HHS (49.3) showed significant improvements (p-value <0.01) in subsequent post-operative follow-ups at six weeks (67.62), one year (95.32) and at the final follow-up (96.17) (Table 2). On doing a Tukey post-hoc analysis, the HHS at six weeks postoperatively when compared to the HHS at one year and at final follow-up also showed significant improvement (p-value<0.01). Although, the HHS at one year and final follow-up did not show any significant difference (p-value 0.86) (Table 3). An example of optimal placement of implants with excellent results is also shown (Figure 2).

**Table 2:** Harris Hip Score at different follow-ups

PARAMETER	INTERVAL	MEAN	SD	ANOVA TEST
HARRIS HIP SCORE	Preop	49.30	6.73	39.27
	6 weeks	67.62	5.27	
	1 year	95.32	4.04	
	Final	96.17	3.78	

**Table 3:** Comparison of Harris Hip Score at different intervals *: statistically significant HSD: honestly significant difference

HARRIS HIP SCORE	TUKEY HSD POST HOC TEST	P VALUE
	Preop vs 6 weeks	<0.01*
	Preop vs 1 year	<0.01*
	Preop vs Final	<0.01*
	6 weeks vs 1 year	<0.01*
	6 weeks vs Final	<0.01*
	1 year vs Final	0.86

The average cup inclination and anteversion angles in the patients were also evaluated on the radiographs at six weeks postoperatively and at the final follow-up. The cup inclination angle and anteversion angle postoperatively and at the final follow-up were 45.08, 47.70 (p-value 0.14) and 19.11, 18.67 (p-value 0.07) respectively with non-significant changes in the values. The LLD was clinically evaluated to assess the shortening in the affected limb (Table 4). There was a significant difference between the pre-operative and six-week postoperative values (2.32cm and 0.25cm respectively; p-value <0.01). However there was no significant difference in the same at six weeks and the final follow-up (0.25cm and 0.49cm respectively) (Table 5).

**Table 4:** Comparison of cup anteversion angle and cup inclination angle between six weeks and final follow up and limb length discrepancy at different follow-ups. *: statistically significant

PARAMETER		MEAN	SD	T TEST	P VALUE
ACETABULAR INCLINATION	6 weeks	45.08	8.59	2.31	0.14
	Final	47.70	8.34		
ACETABULAR ANTEVERSION	6 weeks	19.11	2.98	2.48	0.07
	Final	18.67	1.93		
LIMB LENGTH DISCREPANCY (LLD)	Preop	2.32	0.67	36.59	<0.01*
	6 weeks	0.25	0.41		
	Final	0.49	0.44		

**Table 5:** Comparison of limb length discrepancy at different follow-ups *: statistically significant HSD: honestly significant difference

VARIABLE	TUKEY HSD POST HOC TEST	P VALUE
LIMB LENGTH DISCREPANCY (LLD)	Preop vs 6 weeks	<0.01*
	Preop vs Final	<0.01*
	6 weeks vs Final	0.5

Sixty-six hips showed no signs of osteolysis or migration with a good incorporation of femoral and acetabular components. However, 15 hips showed implant-related complications. Among the complications, nine showed evidence of osteolysis with poly wear and six showed evidence of femoral subsidence (Table 6).

**Table 6:** Occurrence of osteolysis, poly wear and femoral subsidence at different intervals among the study subjects

PARAMETER	INTERVAL	Number of Patients	Percentage (%)
OSTEOLYSIS	1 year	0	0
	Final	9	8.1
POLYWEAR	1 year	0	0
	Final	9	8.1
FEMORAL SUBSIDENCE (AT FINAL FOLLOW-UP)	No	105	94.59
	Yes	6	5.41

**Figure 3:**
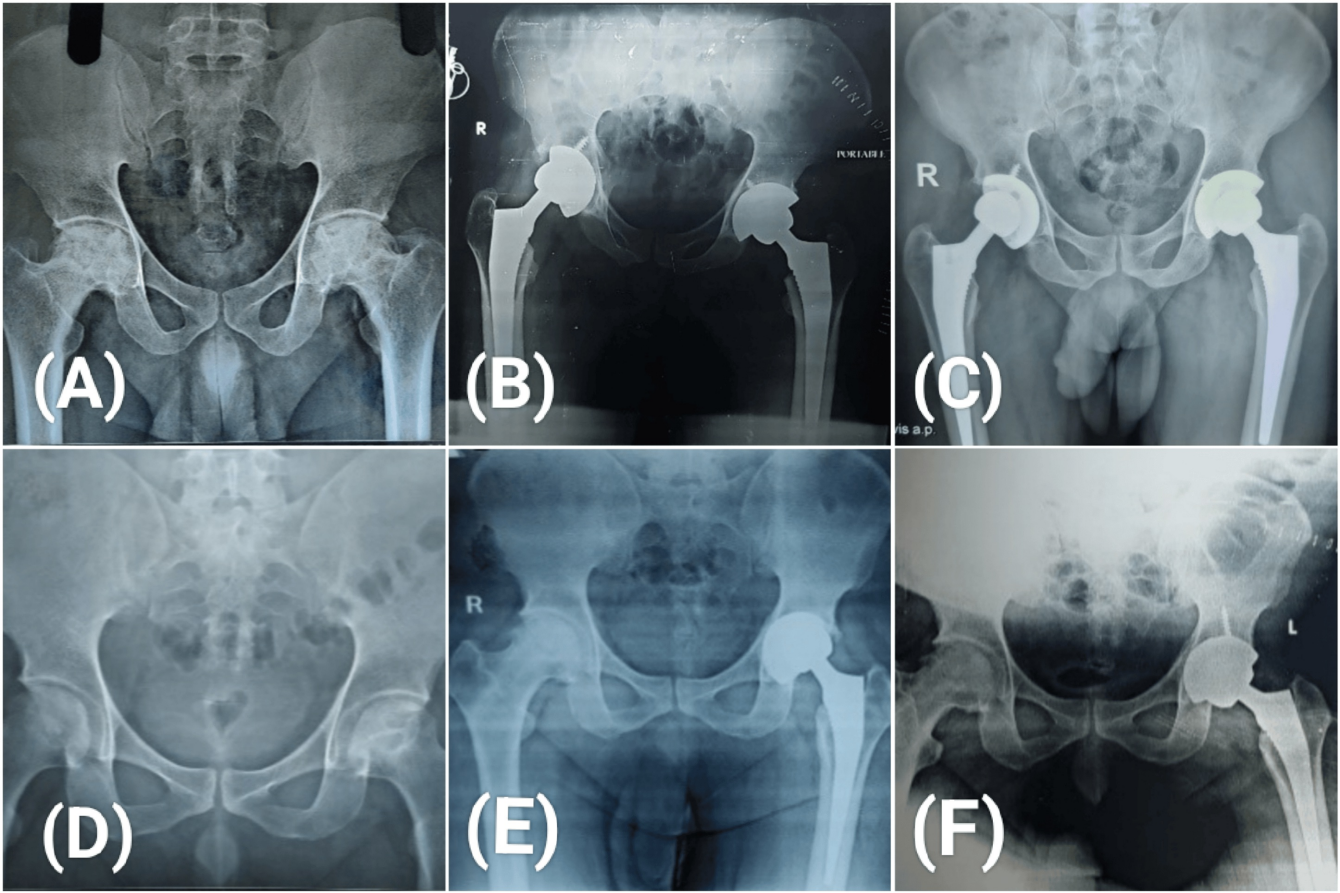
Patient 1- (A) Bilateral Stage Ⅲ ONFH; (B) Post-operative radiograph showing well-placed femoral and acetabular components over left side and high cup abduction angle in the right side; (C) Five-year follow-up showing well-fixed implants with good functional outcome and no reported complication. Patient 2- (D) Bilateral ONFH, Stage ⅡA right side and Stage Ⅲ left side underwent uncemented THA for left side and core decompression for right side; (E) Five-year follow-up radiograph showing low acetabular inclination angle and osteolysis (patient complained of squeaking and instability); (F) Post revision radiograph ONFH: Osteonecrosis of femoral head; THA: Total hip arthroplasty

Of the nine patients with osteolysis, only three showed rates of osteolysis (more than 0.1mm/year) over the acetabular side with cup migration and accelerated eccentric poly wear. These patients underwent revision of the acetabular component (Figure 3).

We also summarized previously done studies that evaluated the demographics, functional outcome and revision rates in patients undergoing THA (Table 7).

**Table 7:** Summary of various studies and their results with comparison of characteristics and outcomes with the present study MoP: Metal on conventional/ultra high molecular weight polyethylene; MoHXLPE: Metal on highly crosslinked polyethylene; CoC: Ceramic on Ceramic

Study	Year of study	Number of Hips	Mean Age (In Years)	Mean Follow Up (In Years)	Bearing Surface	Mean Haris Hip Score		Revision Rate (In %)
						Pre surgery	At final Follow up	
Kim et al [8]	2016	400	52.5	11.8	CoC	45	93	0.5
Philippot et al [9]	2017	137	41	21.9	MoP	52	90	32.1
Stambourgh et al [10]	2016	75	41.2	10	MoHXLPE	46.3	81.9	6.7
Mclaughlin et al [11]	2016	82	36.4	25	MoP	51	89	11
Costa et al [12]	2012	53	20	4.5	MoP	42	93	3.8
Kamath et al [13]	2012	21	18	4.1	CoC, MoHXLPE	43.6	83.6	4.8
Mardani et al [14]	2013	46	24.4	5.2	MoP	59.6	83.5	0
Min et al [15]	2013	162	51.5	7.2	MoHXLPE	48.8	93.4	NR
Present study		111	37.05	4.8	MoP	96.17	-	2.7

## Discussion

Recent trends and studies on ONFH have shown a major preponderance of the disease in the active age group of 20-50 years associated with various etiological factors, the most common being chronic steroid and/or alcohol intake. The majority of these cases present at later stages owing to the general lack of education and awareness, leaving the patients to undergo a major and radical procedure such as THA. Having stated and appreciated the former circumstances, it becomes imperative that the procedure is done so that it can withstand the active and hopefully long-life expectancy of the patient.

Our study showed most patients in the fifth decade (46%). However, the average age of the patients was 37.05 years with a male preponderance (71.43%). Approximately 73% of the patients had grade 4 osteonecrosis according to the Ficat and Arlet classification. Thirty-two percent of patients had bilateral hip involvement. The probable cause of ONFH in most patients was long-term steroid intake (57%) followed by idiopathic (29%) and chronic alcoholism (14%). The baseline characteristics and etiological factors were comparable with studies performed by Vardhan et al., Mclaughlin et al. and Philippot et al. who also found a male preponderance with an average age of in the late third and early fourth decade of life and showed that long-term steroid intake was a major cause of ONFH [1,8]. Extensive use of corticosteroids for more than 12 months makes it an independent variable in the development of ONFH. Steroid intake has been a very common predisposing factor for ONFH in the Indian population, especially in our study region, due to the wide prevalence of quacks and their liberal use of steroids in daily practice.

The functional outcomes were assessed using the HHS which has been proven to have high validity and reliability and is the most widely used scoring system covering domains like hip pain function, motion and deformity [7,9,10]. HHS in our study showed significant improvements from the preoperative values (mean 49.30) to the final follow-up values (mean 96.17). The results of our study were comparable to the results of various other studies that evaluated the short-term outcome in patients who underwent uncemented THA for osteonecrosis of the femoral head with MoP implants and reported a significant increase in mean HHS between preoperative period and the final follow-up [8,11,12].

On the other hand, there have been studies that have evaluated the outcome of ceramic-on-ceramic (CoC) and metal-on-highly crosslinked polyethylene (MoHXLPE) implants in ONFH patients have shown significantly improved HHS scores in the final follow-up [9,10,13,14]. The results of our study were comparable to these studies which shows the durability of MoP implants owing to optimal positioning of the components. 

It has been a long-standing belief that an optimally placed implant is the key to the success of THA. Lewinnek et al. postulated the standard placement of the acetabular components, known as the safe zone, to be 40±10 degrees of cup inclination and 15±10 degrees of anteversion which is supported by numerous established studies worldwide. Acetabular component fixation within this zone reduces the chances of dislocation, instability and early failure to approximately one-fourth of the chances of fixation outside the safe zone [15]. In our present study, the average cup inclination angle was 45.08±8.59 degrees postoperatively and 47.70±8.34 degrees at the final follow-up. Similarly, the mean anteversion was 19.11±2.98 degrees postoperatively and 18.67±1.93 degrees at the final follow-up. Both these values are well within the limits of the safe zone.

Repetitive, high-demand activities in patients cause movement between the bearing surfaces of the implant, causing polyethylene (PE) particle generation and increasing the chances of accelerated polywear and osteolysis. The high wear rate for conventional polyethylene and subsequent osteolysis in the longer term are concerns that must be addressed [16]. As reported by Schmalzier et al., sites of linear and lytic bone losses are filled with macrophages with intracellular polyethylene debris. They found the association of bone resorption with the presence of macrophages laden with polyethylene particulate debris [17]. We have analysed these parameters (on conventional radiograph) and observed stable osseous ingrowth in all components at the last follow-up examination in most of our patients as suggested by no signs of osteolysis at the bone-implant interface. Although, nine of our patients showed minimal linear osteolysis and linear polywear while three patients showed significant osteolysis with cup migration and accelerated eccentric polywear and ultimately landed in failure and underwent acetabular side revision. The lower rate of CPE wear in our stable and precisely fixed components could also be the reason for the excellent results.

Vincent et al. in their conceptual review stated that postoperative lengthening of 1 cm or shortening of 0.35 cm over the operated side was acceptable in patients who underwent THA [18]. However, there is no strict parameter to define an acceptable LLD since the difference in limb lengths is usually compensated by the pelvic obliquity and/or functional adjustments in the ipsilateral knee and ankle joints. The mean pre-operative LLD in the affected limb in patients of our study was 2.32 cm which was managed with proper preoperative planning and templating with a reduction to 0.25 cm postoperatively. An insignificant increase in LLD to 0.49 cm was noticed at the final follow-up which can be attributed to an impaction at the implant-bone interface and probably due to minimal acceptable femoral subsidence. Cinotti et al. conducted a study to evaluate femoral subsidence in uncemented THA patients. They reported a mean femoral subsidence of 2.2 mm which was the maximum in the initial six to eight weeks. This was probably due to the biological stability and osteointegration which was more evident until the initial two months of surgery while inadequate cancellous bone impaction intraoperatively could lead to subsidence in the short term. Morrey et al. postulated that the rates of revision in THA patients were significant in the patients with femoral subsidence of more than 4 mm. The average subsidence among the patients in their study was 2.5 mm [10,19]. We have reported six patients with significant femoral subsidence (>4 mm). However, all these patients with significant femoral stem subsidence are doing well with no complaints. The other few complications we encountered in early post-operative periods were 12 patients with hip and thigh pain which were managed effectively with analgesics (nonsteroidal anti-inflammatory drugs (NSAIDs)) and three patients with surgical site infection who were successfully managed with debridement, antibiotics, irrigation, and implant retention (DAIR).

Mei et al. reviewed 32 studies reporting 3219 primary THA in 2434 patients with the most common aetiology being ONFH. 2214 (68.8%) hips were cementless, 540 (16.8%) hybrid and 465 (14.4%) cemented [20]. The five-year survival rate in their study was 98.7% and the rates of major complications like dislocation and deep infection were 2.4%. The five-year survival rate in our study was 97.29% and the rates of infection were 2.7% with no incidence of dislocation or gross instability. The revision surgeries in our study were attributed to an inappropriately placed acetabular component which led to accelerated poly wear. All the remaining hips in our study are performing well to date and are subject to further follow-up.

The majority of the patients in our described study had labour-intensive occupations which needed high functional demands over long durations of physical work and still showed a near-complete return to prior occupational ability and normal activities of daily living, which proves that well-placed implants following the well-laid-out and extensively researched principles of total hip arthroplasty were able to provide these patients good functional and radiological outcomes.

Our study had a few limitations. This was a single-centre study having a relatively small follow-up period and a small sample size with the absence of a control group. The choice of implant was affected by the implant allowance in the surgical packages provided under the funded schemes. However, our findings remain valuable and relevant because the study focused on a group of consecutive patients with the same diagnosis who were treated by a single surgeon using the same type of implant and showed excellent mid-term follow-up results.

## Conclusions

Our mid-term follow-up of the recruited patients, mainly of the labour class, has shown promising and encouraging results in terms of return to heavy and physically demanding activities, however, a longer follow-up for further information is warranted. Unsolicited, careless and long-term use of corticosteroids is emerging as a potential risk factor for the development of osteonecrosis of the femoral head in the Indian population and has to be curtailed and curated. The patients in our study showed excellent functional outcomes with a safe and durable fixation providing satisfactory ranges of stable motion and very few complications. The findings of our study can be built upon by comparing them to the outcomes following the use of newer implants and their efficacy in different age groups.
